# P[8] and P[4] Rotavirus Infection Associated with Secretor Phenotypes Among Children in South China

**DOI:** 10.1038/srep34591

**Published:** 2016-10-06

**Authors:** Xu-Fu Zhang, Yan Long, Ming Tan, Ting Zhang, Qiong Huang, Xi Jiang, Wen-Fang Tan, Jian-Dong Li, Gui-Fang Hu, Shixing Tang, Ying-Chun Dai

**Affiliations:** 1 School of Traditional Chinese Medicine, Southern Medical University, Guangzhou, Guangdong, China; 2 Divisions of Infectious Diseases, Cincinnati Children’s Hospital Medical Center, Cincinnati, Ohio, USA; 3 Department of Pediatrics, University of Cincinnati College of Medicine, Cincinnati, Ohio, USA; 4 Department of Epidemiology, School of Public Health, Southern Medical University (Guangdong Provincial Key Laboratory of Tropical Disease Research), Guangzhou, Guangdong, China; 5 Guangdong Provincial Center for Disease Control and Prevention, Guangzhou, China

## Abstract

Rotaviruses are known to recognize human histo-blood group antigens (HBGAs) as a host ligand that is believed to play an important role in rotavirus host susceptibility and host range. In this study, paired fecal and saliva samples collected from children with viral gastroenteritis, as well as paired serum and saliva samples collected from the general population in south China were studied to evaluate potential association between rotavirus infections and human HBGA phenotypes. Rotavirus was detected in 75 (28%) of 266 fecal samples and P[8] rotaviruses were found to be the predominant genotype. The HBGA phenotypes of the rotavirus-infected children were determined through their saliva samples. Secretor statuses were found to correlate with the risk of rotavirus infection and all P[8]/P[4] rotavirus infected children were secretors. Accordingly, recombinant VP8* proteins of the P[8]/P[4] rotaviruses bound saliva samples from secretor individuals. Furthermore, correlation between serum P[8]/P[4]-specific IgG and host Lewis and secretor phenotypes has been found among 206 studied serum samples. Our study supported the association between rotavirus infection and the host HBGA phenotypes, which would help further understanding of rotavirus host range and epidemiology.

Group A rotaviruses are the leading cause of severe, epidemic gastroenteritis in infants and young children worldwide, in which P[8] and P[4] are usually the first and the second predominant genotypes, respectively in causing rotavirus diarrhea. Prior to the introduction of rotavirus vaccines, rotaviruses infected 130 million children, resulting in two million hospitalizations, and about 450,000 deaths globally every year^1^. The numbers of rotavirus related illness and death have significantly declined with, for example, 215,000 deaths in 2013^2^,^3^, as a result of the use of two effective rotavirus vaccines, Rotarix (GlaxoSmithKline) and RotaTeq (Merck), since 2006. However, neither Rotarix nor RotaTeq has been licensed in China and a lamb rotavirus based vaccine [Rotavirus (Live) Vaccine, Oral] that was developed in China has not yet been widely used among Chinese children^4^. As a consequence, a high mortality attributed to rotavirus infection remains in China that was estimated to be ~42% of all deaths from diarrhea^5^.

While our current understanding of the initial stage of rotavirus infection and the viral receptors or ligands remains limited, recent studies showed that attachments of major human rotaviruses to their host cells to initiate an infection may be mediated by histo-blood group antigens (HBGAs)^6^,^7^,^8^. HBGAs are complex carbohydrates that distribute abundantly on the mucosal epithelia of the gastrointestinal tracts. Rotaviruses recognize the HBGAs on host cell surface through the VP8 subdomain of the spike protein VP4, resulting in attachments of rotaviruses to host cells in P genotype-dependent manner. Therefore, HBGAs may function as the host ligands or receptors playing an important role in rotavirus infection and host susceptibility^6^,^7^,^8^, although other factors may also have a role in the infection and host susceptibility of rotaviruses. In fact, an association of P[8] rotaviruses infection with the secretor statuses of human hosts has been observed^9^,^10^,^11^,^12^, matching the HBGA binding pattern that P[8] rotaviruses recognize the Le^b^ and H type 1 antigens^7^. However, a study performed among Tunisian children showed that P[8] rotaviruses infected both secretor and non-secretor individuals^13^, which appeared to be conflicted with the above observed association. This discrepancy indicates the necessity for further population-specific studies to understand the role of host HBGA phenotypes in rotavirus infection and host ranges.

In this study, we performed an epidemiologic investigation, focusing on children with acute gastroenteritis visiting a large hospital in Guangzhou of south China. We found that P[8] and P[4] rotavirus infections correlated with secretor statuses of the children. Accordingly, the recombinant VP8* proteins of P[8] and P[4] rotaviruses bound saliva samples from secretor individuals. In addition, an association between P[8]/P[4] rotavirus-specific IgG antibody titers and their Lewis and secretor statuses were also observed in the general population of similar area of China.

## Results

### P[8] genotype was predominant during the study period

Of the 266 patients with diarrhea visiting Nanfang Hospital, 75 had confirmed rotavirus infections, among which 61 (81.3%) were children under two-years of age. As expected, vast majority (70) of the detected rotaviruses belonged to P[8], while the remaining five belonged to P[4] genotypes. This observed predominance was typical, similar like what was observed in other regions of China^14^,^15^.

### HBGA phenotyping of the rotavirus-infected individuals

**Figure 1:**
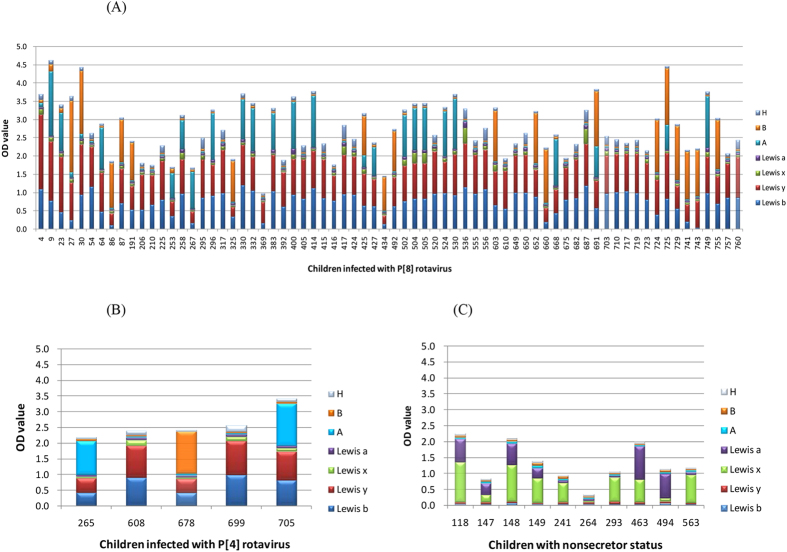
The A/B/H and Lewis antigen profiles of the saliva samples from children who was infected with P[8] (**A**) or P[4] (**B**) rotavirus infection. The histo-blood group antigen (HBGA) profiles of the ten nonsecretors were shown in (**C**).The numbered saliva samples are indicated in the X-axis. Different HBGAs are shown in various colors as indicated.

The A, B, H, Le^a^, Le^b^, Le^x^, and Le^y^ antigens of the saliva samples of the 75 children with rotavirus infection were determined by EIA using specific monoclonal antibodies for HBGA phenotyping (Fig. 1). All 75 rotavirus-infected children were found to be secretors without a single non-secretor. In addition, we determined the HBGA phenotypes of the children with diarrhea who were rotavirus and norovirus negative as internal negative controls. Although similar distribution patterns of A/B/O blood types among groups of the rotavirus-infected individuals, the internal negative controls, and the healthy controls were seen (Table 1), their Lewis and secretor statuses are clearly different (see below).

### Lewis (Le) and secretor statuses associated with the risk of rotavirus infection

While there was no statistic difference in the distributions of A/B/O blood types between the rotavirus-infected individuals and those in the internal negative control group (*P* = 0.384), further comparison of the two groups indicated a significant lower proportion of Le^a+^/Le^x+^ (*P* = 0.004), higher proportion of Le^a+b+^/Le^x+y+^ (*P* = 0.004), and lower proportion of non-secretor (*P* = 0.033) statuses in the rotavirus-infected children (Table 1). These data indicated that the host Lewis and secretor statuses correlated with the risk of rotavirus infection.

Odds ratio analysis revealed similar associations between HBGAs and the risks of rotavirus infection. When compared with internal control group, the secretor individuals were found at significantly higher infection risk than the non-secretors (OR = 1.531, 95% CI 1.39–1.69 vs. OR = 0.653, 95%). Significantly higher infection risk was also found among those with Le^a+b+^ or Le^x+y+^ (OR = 3.005, 95% CI 1.205–7.494) and those with Le^a+^ or Le^x+^ (OR = 0.653,CI 0.592–0.719) (Table 1). In contrast, no significant difference regarding the risk of rotavirus infections was found among individuals with different A, B, and O secretor bloodtypes (Table 1).Similar results of HBGA distribution and risk were also showed when compared with healthy control group. However, no correlation was found between secretor status and Lewis phenotypes with clinical symptoms (data not shown).

### P[8] and P[4] rotavirus VP8* proteins bound saliva samples from secretor donors

**Figure 2:**
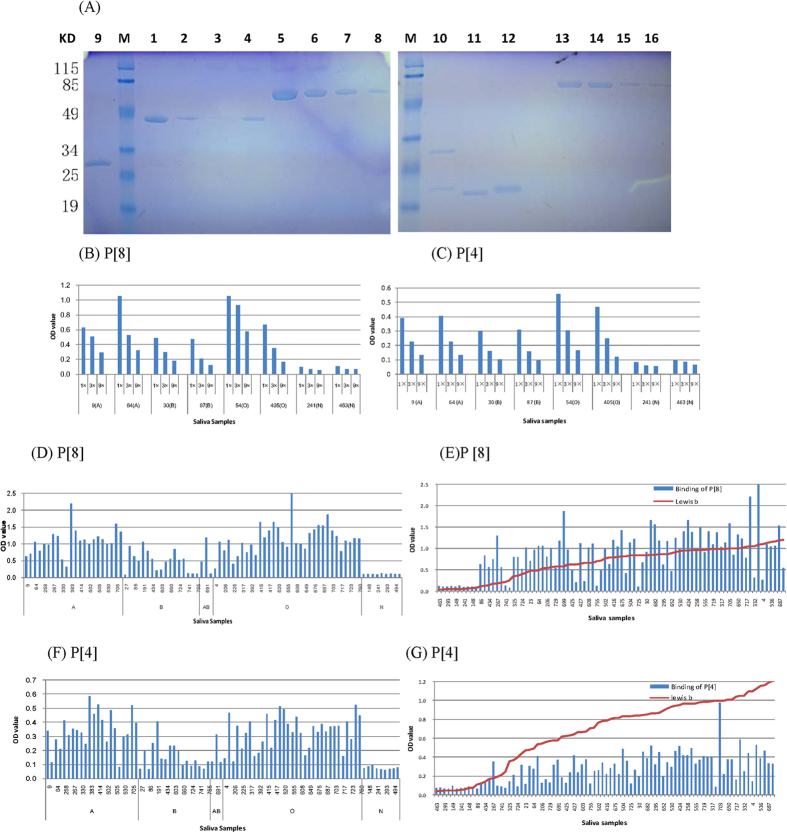
Production of the GST-VP8* fusion proteins and their saliva-based HBGA bindings. (**A**) Analysis of the GST-VP8* fusion proteins of P[8] and P[4] rotaviruses by SDS-PAGE with bovine serum albumin (BSA) as standards on the same gels for protein quantitation. Lane 1–3: elution fraction 1 to 3 of the GST-VP8* fusion protein of P[8] rotavirus; lane 4: elution 1 of the GST-VP8* fusion protein of P[4] rotavirus; lane 5–8: BSA standard protein at amounts of 8, 4, 2, 1 μg, respectively; lane 9: VP8* protein of P[8] rotavirus; lane 10: VP8* protein of P[4] rotavirus; lane 11–12: GST elution; lane 13–16: BSA standard protein at amounts of 4, 3, 2, 1 μg, respectively; M: protein marker. (**B**,**C**) Eight boiled saliva samples were coated onto 96-well plates and then incubated with the GST-VP8* fusion proteins of P[8] (**B**) or P[4] (**C**) rotavirus. The protein was tested in a series of 3-fold dilutions (15 μg/ml, 5μg/ml and 1.7 μg/ml) by enzyme-linked immunosorbent assay (ELISA). (**D–G**) The binding activities of the GST-VP8* fusion proteins of P[8] (**D**,**E**) or P[4] (**F**,**G**) rotavirus at 10 μg/ml to 85 saliva samples, among which 75 were from individuals with confirmed rotavirus infection, while 10 were from non-secretor individuals without rotavirus infection. The saliva samples were sorted by blood types (**D**,**F**), or by the Leb signals of individual saliva samples (**E**,**G**). “A,” “B,” “O,” and “N” represent the type A, B, O and non-secretor saliva, respectively.

Both GST-VP8* fusion proteins of P[8] and P[4] rotaviruses were expressed well through the *E. coli* expression system (Fig. 2A). Saliva-based HBGA binding assays showed that both fusion proteins bound the saliva samples of type A, B, and O secretors that were also Le^b^ antigen positive in a dose-dependent manner (Fig. 2B,C), but not the saliva samples from non-secretors that were Le^b^ antigen negative, similar to what was observed in a previous study^7^. The saliva samples collected from the rotavirus-infected individuals of this study also bound well to the fusion proteins (Fig. 2D,E). These binding data were consistent with the observed host susceptibility correlated with host secretor status. In addition, we found that the binding signals of P[4] VP8* to secretor saliva samples were weaker than those of P[8] VP8* (Fig. 2D,E), probably explaining the lower prevalence of P[4] rotavirus compared with that of P[8] rotavirus in this study, although other unknown factors may also contribute to this lower prevalence.

### Serum P[8]/P[4] rotavirus IgG was correlated with host Lewis and secretor phenotypes

**Figure 3:**
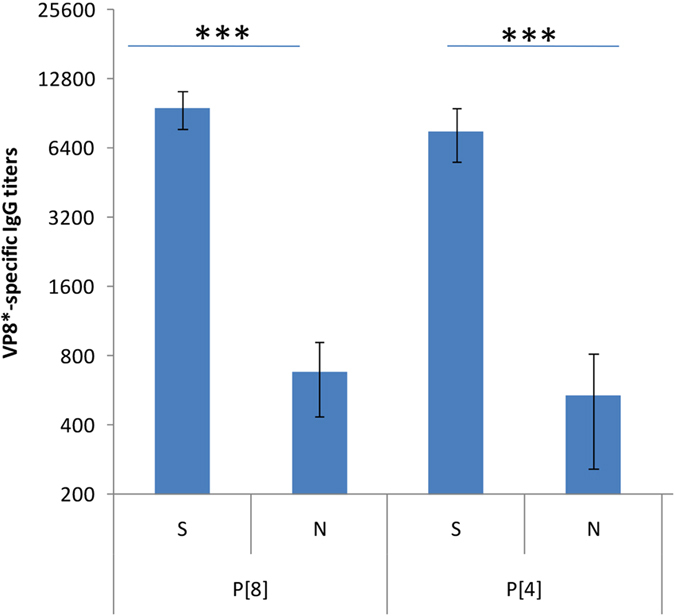
The geometric means of P[8]/P[4] rotavirus VP8-specific antibody titers (Y-axis) in the secretor (S) and non-secretor (N) individuals. The error bars shows standard error. **P < 0.001.

To further understand the observed association between the host HBGA phenotypes and rotavirus infection, we determined the P[8] and P[4] rotavirus-specific IgG antibody titers and their HBGA phenotypes based on 206 paired serum and saliva samples collected from the general population of Shanwei city, another region of Guangdong province (Table 2). Compared with the individuals with negative P[8] rotavirus-specific IgG, a significant higher proportion of secretor (90.2% vs 78.1%, P = 0.027) but a lower proportion of Le^a+^/Le^x+^ (9.9% vs 21.9%, P = 0.001) were found among the individuals with positive P[8] rotavirus-specific IgG. These results showed that the Le^a+^/Le^x+^ and the non-secretor statuses of the individual correlated negatively with the proportion of positive P[8] antibody of the individuals (*Ps* < 0.05). In contrast, A/B/O phenotypes appeared not to show such association (Table 2). Similar scenario were also seen for P[4] rotavirus IgG antibody (Table 2). Further analysis showed that, the mean antibody titers of P[8] and P[4] among secretor were significantly higher than those of non-secretor (P < 0.001, Fig. 3), consistent with the above observed susceptibility of the two rotaviruses.

## Discussion

A number of recent reports demonstrated that human rotaviruses recognize the HBGA carbohydrates in a P type-specific manner^6^,^7^,^8^,^16^,^17^,^18^, a phenomenon like what was observed in human noroviruses^19^,^20^,^21^.The HBGA binding sites of rotavirus have been identified on the VP8*, the head of the spike protein VP4^6^,^8^, making the VP8* protein an excellent model for study of rotavirus-HBGA interaction. The biological significance of such interaction in rotavirus host susceptibility was partially shown by the observation of the association between the host HBGA phenotypes and their risks of rotavirus infection^9^,^10^,^11^. So far evidence supporting this notion included epidemiology studies that were performed in different continents/countries, including North America (USA), Europe (France and Sweden), Asia (China and Vietnam), and Africa (Burkina Faso)^9^,^10^,^11^,^12^,^22^,^23^, pointing to the same conclusion that P[8] rotaviruses infected only secretor children, but not non-secretor individuals. These observed host susceptibilities are consistent with the HBGA binding profile of the P[8] VP8* protein to the Le^b^ and H type 1 antigens, the HBGAs that are commonly present among the secretor individuals, but is absent in the non-secretors^21^. This present study adds further support to this notion, although discrepancy was found in a separate study on Tunisia children, which revealed P[8] rotaviruses infecting both secretors and non-secretors^13^. Future study will be necessary to clarify this issue.

In addition to the correlation between the infection risk and the host HBGA phenotypes, we also investigated the relationship between the P[8]/P[4] rotavirus-specific antibodies in the serum samples of the general Chinese population and their HBGA phenotypes. As expected, we found that individuals with matched Lewis and secretor phenotypes exhibited significantly higher proportion of serum P[8]/P[4] rotavirus antibodies than those with unmatched HBGAs. By contrast, there was no such correlation between the antibody titers and the A/B/H antigens. These data further support the Le^b^ and secretor statuses as an important host susceptible factor for the infection of P[8]/P[4] rotaviruses.

P[8] and P[4] are two common rotavirus genotypes causing more than 90% cases of rotavirus-associated diarrhea in many countries including China^24^,^25^. Thus, it is not surprising to see all detected rotaviruses in this present study belong to these two genotypes. Both P[8] and P[4] rotaviruses have been shown to bind Le^b^ and H type 1 antigens that are presenting widely in the secretor individuals of the general population. This wide distribution of the Le^b^ antigen may be an important reason for the high prevalence of two rotavirus genotypes.

## Methods

### Samples collection

Paired feces and saliva samples were collected from 266 children <5 years of age with acute viral gastroenteritis who visited pediatrics outpatient department at Nanfang Hospital in Guangzhou, Guangdong province, China, from November of 2013 to January of 2015. Saliva samples collected from 159 children in a primary school in the same area were used as healthy control. The study was approved by the Ethics Committee of the Nanfang Hospital, Southern Medical University (No. NFHEC-2013.310). Informed consents were obtained from parents of all study participants.

In addition, paired serum and saliva samples were collected from 206 individuals of the general population at ages from 3–88 year old with a median age of 51.2 in Magong and Hongsao districts in Shanwei city of Guangdong province, China, during January 24 to 27, 2015. The study was approved by the Ethics Committee of CDC of Guangdong province (No. GDCDC-W96-027B-2014.101). Informed consents were obtained from participants. 10–50 μl saliva was collected using sterile plastic micropipette under tongue from each person. All sample collection and the downstream experiments were performed in accordance with relevant guidelines and regulations. Study population demographics were shown in supplement Table 1.

### Detection of rotaviruses and their genotyping

RNA was extracted from 10% feces suspension using TRIzol Reagent (Invitrogen, Carlsbad, CA, USA). Rotavirus was detected by RT-PCR using a pair of VP4 gene specific primers (VP4F and VP4R); and the P types of the detected rotaviruses were then determined using VP4F combined with P-typing reverse primer 1T1 (P[8]), 2T1 (P[4]), 3T1(P[6]), 4T1 (P[9]) and 5T1 (P[10]) as described previously^26^. Randomly selected typed and all untyped samples were sequenced in Invitrogen Company (GZ, CN). Norovirus was also examined by RT-PCR using primers GI-SKF/GI-SKR and COG-2F/GII-SKR as described previously^27^.

### HBGA phenotyping of the studied individuals

Various HBGA antigens of the saliva samples were determined by EIA assays using the corresponding monoclonal antibodies specific for A (Z2A), B (Z5H-2), H (87-N) (Santa Laboratories, Inc., CA), Lewis a (Le^a^, BG-5), Lewis b (Le^b^, BG-6), Lewis X (Le^x^, BG-7), and Lewis y (Le^y^, BG-8) antigens (Signet Dedham, MA) as described previously^28^. The cut-off of a positive signal was OD_450_ = 0.15, which was an approximate value of the mean of the background/blank wells plus three times the standard deviation. Saliva samples from Children who were rotavirus negative were grouped as a negative control for comparison with the rotavirus infection group. Saliva samples collected from 159 children in a primary school in the same area were used as healthy control.

### Production of recombinant rotavirus VP8* proteins

Previous studies showed that the GST-VP8* is an excellent tool to study rotavirus-HBGA interaction^6^,^7^,^17^. The recombinant GST-VP8*fusion proteins of a P[8] (isolate #23) and a P[4] (isolate #705) rotavirus isolated were expressed *E. coli* and purified for saliva-based HBGA binding assay as described previously^7^. The DNA fragments encoding VP8* with 185 residues [residue 49 to 233numbered according to WA (GenBank AC#: VPXRWA)] of the P[8] and P[4] rotaviruses from the corresponding stool samples were cloned into plasmid pGEX-4T-1 (GST Gene Fusion System, GE Healthcare Life Sciences) via the Bam HI and Sal I sites. To obtain free VP8* proteins for specific rotavirus IgG determination (see below), GST-VP8* fusion proteins were digested by thrombin (GE Healthcare life Sciences, NJ, USA)at room temperature overnight, while fusion protein still bound to the purification resin. The free VP8* was then released by elution.

### SDS-PAGE and protein quantitation

Quality of the purified proteins were evaluated by SDS-PAGE using 10% separating gels. The GST-VP8* fusion and the VP8* proteins were quantitated by SDS-PAGE using serially diluted bovine serum albumin (BSA, Tiangen Biocomany, CN) as standards on same gels. The gels were stained with Page Blue Protein Staining Solution (Tiangen Biocomany, CN). Through comparison of the signal insensitivities with the BSA with known concentration, the concentrations of the GST-VP8* fusion and the VP8* proteins were estimated. After quantitation, the proteins were aliquoted for VP8*-HBGA binding assay.

### VP8*-HBGA binding assay

Diluted saliva samples (1:1000) were coated onto 96-well microtiter plates (Grenier Inc.) overnight at 4 °C. After blocking with 5% nonfat milk, GST-VP8* protein at 10 μg/ml was added and incubated for 1 hour at 37 °C. The bound VP8* proteins were detected using our in-house made rabbit serum anti-rotavirus antibodies (1:3000)^7^. Horseradish peroxidase (HRP)-conjugated goat anti-rabbit IgG (1:2000, Abcam, Cambridge, UK) was used as the secondary antibody. The signal intensities were displayed using a TMB kit (Beyotime Biotechnology Co., Ltd, Shanghai, CN) and the optical density (OD) at 450 nm was read. The cut-off for a positive binding signal was OD450 = 0.15, which was an approximate value of the mean of the background/blank wells plus three times the standard deviation.

### Determination of Rotavirus P genotype-specific IgG antibodiesin human sera

This was measured by EIA using P genotype-specific VP8* proteins as capture antigens. Free VP8* proteins at 0.5 μg/ml in phosphate buffer saline (PBS, pH 7.4) were coated on 96-well plates (Grenier Inc.) at 4 °C overnight. After blocked with 5% nonfat dry milk at 37 °C for 1 hr, human serum samples in two-fold serial dilution in 5% nonfat dry milk were added to the plates and incubated for1hour at 37 °C. HRP-conjugated goat anti-human IgG (1:5000, Abcam clonal Biotechnology, Co., Ltd, US) in 5% nonfat dry milk was used to detect the bound human IgG. The bound HRP signals were displayed using a TMB substrate kit (Beyotime Biotechnology Co., Ltd, Shanghai, CN). Human sera with positive rotavirus IgG were defined as EIA signals >0.2 at 800-fold dilution of the serum samples.

### Statistical analysis

The statistical difference between data groups were analyzed through the Fisher exact test with 2-tailed. Unadjusted odds ratios (OR) and 95% confidence intervals (CIs) were calculated using software Statistical Product and Service Solutions (SPSS) 20.0 for Windows 7 (SPSS Inc., Chicago, IL, USA).

## Additional Information

**How to cite this article**: Zhang, X.-F. *et al*. P[8] and P[4] Rotavirus Infection Associated with Secretor Phenotypes Among Children in South China. *Sci. Rep.* **6**, 34591; doi: 10.1038/srep34591 (2016).

## Supplementary Material

Supplementary Information

## Figures and Tables

**Table 1:** Associations and Influence of A/B/O, Lewis, and secretor blood types among children with the risk of rotavirus infection.

Status	No. (%) of RV positive	No. (%) of RV/NoV^†^ negative	OR (95% CI), *P value*	No. (%) of health control	OR (95% CI), *P value*
Blood type	n = 75	n = 151	**N** **=** **226, ^P = 0.384**	n = 159	**N** = **234, ^P** = **0.672**
A	22 (29.3)	47 (31.1)	**0.919 (0.502–1.682),** ***0.878***	35 (22.0)	**1.471 (0.789–2.741),*****0.254***
B	17 (22.7)	44 (29.1)	**0.713 (0.374–1.358),** ***0.324***	39 (24.5)	**0.902 (0.471–1.728),** ***0.870***
O	33 (44.0)	50 (33.1)	**1.587 (0.899–2.801),** ***0.143***	79 (49.7)	**0.796 (0.458–1.382),*****0.484***
AB	3 (4.0)	10 (6.6)	**0.588 (0.157–2.202),*****0.552***	6 (3.8)	**1.063 (0.258–4.369),** ***1.000***
Lewis status	n = 75	n = 151	**N = 226, ^P** = 0.004**	n = 159	**N** **=** **234,** ^**^**^**P** **=** 0.000**
Le^b+^/Le^y+^	63 (84.0)	132 (87.4)	**1.198 (0.673–1.21),** ***0.539***	134 (84.3)	**0.979 (0.462–2.075),** ***1.000***
Le^a+^/Le^x+^	0 (0.0)	10 (5.6)	**0.653 (0.592–0.719),** 0.033*	20 (12.6)	**0.874 (0.824–0.927),** 0.001**
Le^a+b+^/Le^x+y+^	12 (16.0)	9 (6.0)	**3.005 (1.205–7.494),**0.026*	5 (3.1)	**5.869 (1.985–17.339),** 0.001**
Secretor status^§^	n = 75	n = 151	**N** **=** **226, ^P** **=** 0.033*	n = 159	**N** **=** **234, ^P** **=** 0.001**
Secretor	75 (100)	141 (93.4)	**1.531 (1.39–1.69),**0.033*	139 (87.4)	**1.144 (1.079–1.214),** 0.001**
Nonsecretor	0 (0)	10 (6.6)	**0.653 (0.592–0.719)**, 0.033*	20 (12.6)	**0.874 (0.824–0.927),** 0.001**

^†^NoV samples were excluded from patients without RV infection; Unadjusted odds ratios (OR) and 95% confidence intervals (CIs); ^§^Le^b+^/Le^y+^ and Le^a+b+^/Le^x+y+^ were grouped into secretor and Le^a+^/Le^x+^ were grouped into nonsecretor; ^**^**^P: significance test of proportion of different HBGA phenotype between RV positive group and control group; *P < 0.05; **P < 0.01.

**Table 2:** Association between rotavirus IgG antibodies and the A/B/O, Lewis and secretor bloodtypes.

Status	P[8]		P value	P[4]		P value
	No. (%) of positive	No. (%) of negative		No. (%) of positive	No. (%) of negative	
Blood type	142	64	0.677	134	72	0.157
A	31 (21.8)	16 (25.0)		31 (23.1)	16 (22.2)	
B	32 (22.5)	11 (17.2)		33 (24.6)	10 (13.9)	
O	74 (52.1)	36 (56.2)		65 (48.5)	45 (62.5)	
AB	5 (3.5)	1 (1.6)		5 (3.7)	1 (1.4)	
Lewis status	142	64	0.001**	134	72	0.000**
Le^b+^/Le^y+^	118 (83.1)	38 (59.4)		114 (85.1)	42 (58.3)	
Le^a+^/Le^x+^	14 (9.9)	14 (21.9)		11 (8.2)	17 (23.6)	
Le^a+b+^/Le^x+y+^	10 (7.0)	12 (18.8)		9 (6.7)	13 (18.1)	
Secretor status^§^	142	64	0.027*	134	72	0.005**
Secretor	128 (90.2)	50 (78.1)		123 (91.8)	55 (76.4)	
Nonsecretor	14 (9.8)	14 (21.9)		11 (8.2)	17 (23.6)	

^§^Le^b+^/Le^y+^ and Le^a+b+^/Le^x+y+^ were grouped into secretor and Le^a+^/Le^x+^ were grouped into nonsecretor; *P < 0.05; **P < 0.01.

## References

[b1] Tate J. E. . 2008 estimate of worldwide rotavirus-associated mortality in children younger than 5 years before the introduction of universal rotavirus vaccination programmes: a systematic review and meta-analysis. The Lancet. Infectious diseases 12, 136–141 (2012). 22030330 10.1016/S1473-3099(11)70253-5

[b2] Lopman B. A. . Post-licensure experience with rotavirus vaccination in high and middle income countries; 2006 to 2011. Current opinion in virology 2, 434–442 (2012). 22749491 10.1016/j.coviro.2012.05.002

[b3] Tate J. E., Burton A. H., Boschi-Pinto C. & Parashar U. D. World Health Organization-Coordinated Global Rotavirus Surveillance N. Global, Regional, and National Estimates of Rotavirus Mortality in Children <5 Years of Age, 2000–2013. Clin Infect Dis 62 Suppl 2, S96–S105 (2016). 27059362 10.1093/cid/civ1013 PMC11979873

[b4] Liu N. . Update on the disease burden and circulating strains of rotavirus in China: a systematic review and meta-analysis. Vaccine 32, 4369–4375 (2014). 24958704 10.1016/j.vaccine.2014.06.018

[b5] Zhang J. . Rotavirus-specific and Overall Diarrhea Mortality in Chinese Children Younger than 5 Years: 2003 to 2012. The Pediatric infectious disease journal 34, e233–237 (2015). 26083587 10.1097/INF.0000000000000799 PMC4618544

[b6] Hu L. . Cell attachment protein VP8* of a human rotavirus specifically interacts with A-type histo-blood group antigen. Nature 485, 256–259 (2012). 22504179 10.1038/nature10996 PMC3350622

[b7] Huang P. . Spike protein VP8* of human rotavirus recognizes histo-blood group antigens in a type-specific manner. J Virol 86, 4833–4843 (2012). 22345472 10.1128/JVI.05507-11 PMC3347384

[b8] Hu L. . Structural basis of glycan specificity in neonate-specific bovine-human reassortant rotavirus. Nat Commun 6, 8346 (2015). 26420502 10.1038/ncomms9346 PMC4589887

[b9] Van Trang N. . Association between norovirus and rotavirus infection and histo-blood group antigen types in Vietnamese children. Journal of clinical microbiology 52, 1366–1374 (2014). 24523471 10.1128/JCM.02927-13 PMC3993640

[b10] Imbert-Marcille B. M. . A FUT2 gene common polymorphism determines resistance to rotavirus A of the P[8] genotype. The Journal of infectious diseases 209, 1227–1230 (2014). 24277741 10.1093/infdis/jit655

[b11] Nordgren J. . Both Lewis and secretor status mediate susceptibility to rotavirus infections in a rotavirus genotype-dependent manner. Clin Infect Dis 59, 1567–1573 (2014). 25097083 10.1093/cid/ciu633 PMC4650770

[b12] Kambhampati A., Payne D. C., Costantini V. & Lopman B. A. Host Genetic Susceptibility to Enteric Viruses: A Systematic Review and Metaanalysis. Clin Infect Dis 62, 11–18 (2016). 26508510 10.1093/cid/civ873 PMC4679673

[b13] Ayouni S. . Rotavirus P[8] Infections in Persons with Secretor and Nonsecretor Phenotypes, Tunisia. Emerging infectious diseases 21, 2055–2058 (2015). 26488868 10.3201/eid2111.141901 PMC4622234

[b14] Xie X. L. . Molecular epidemiology of viral diarrhea in Chengdu infants and young children. Zhonghua shi yan he lin chuang bing du xue za zh = Zhonghua shiyan he linchuang bingduxue zazhi = Chinese journal of experimental and clinical virology 26, 2–4 (2012). 22919739

[b15] Wang Y. H. . Molecular epidemiology and genetic evolution of the whole genome of G3P[8] human rotavirus in Wuhan, China, from 2000 through 2013. PloS one 9, e88850 (2014). 24676363 10.1371/journal.pone.0088850 PMC3967987

[b16] Liu Y. . Poly-LacNAc as an age-specific ligand for rotavirus P[11] in neonates and infants. PLoS One 8, e78113 (2013). 24244290 10.1371/journal.pone.0078113 PMC3823915

[b17] Liu Y. . Rotavirus VP8*: phylogeny, host range, and interaction with histo-blood group antigens. J Virol 86, 9899–9910 (2012). 22761376 10.1128/JVI.00979-12 PMC3446626

[b18] Ramani S. . The VP8* domain of neonatal rotavirus strain G10P[11] binds to type II precursor glycans. J Virol 87, 7255–7264 (2013). 23616650 10.1128/JVI.03518-12 PMC3700318

[b19] Tan M. & Jiang X. Norovirus gastroenteritis, carbohydrate receptors, and animal models. PLoS pathogens 6, e1000983 (2010). 20865168 10.1371/journal.ppat.1000983 PMC2928792

[b20] Tan M. & Jiang X. Norovirus-host interaction: Multi-selections by human histo-blood group antigens. Trends in microbiology 19, 382–388 (2011). 21705222 10.1016/j.tim.2011.05.007 PMC3149758

[b21] Tan M. & Jiang X. Histo-blood group antigens: a common niche for norovirus and rotavirus. Expert Rev Mol Med 16, e5 (2014). 24606759 10.1017/erm.2014.2 PMC12406300

[b22] Payne D. C. . Epidemiologic Association Between FUT2 Secretor Status and Severe Rotavirus Gastroenteritis in Children in the United States. JAMA pediatrics 169, 1040–1045 (2015). 26389824 10.1001/jamapediatrics.2015.2002 PMC4856001

[b23] Ma X. . Binding Patterns of Rotavirus Genotypes P[4], P[6], and P[8] in China with Histo-Blood Group Antigens. PloS one 10, e0134584 (2015). 26274396 10.1371/journal.pone.0134584 PMC4537235

[b24] Mayindou G. . Molecular epidemiology and surveillance of circulating rotavirus and adenovirus in Congolese children with gastroenteritis. J Med Virol 88, 596–605 (2016). 26378607 10.1002/jmv.24382

[b25] Sai L. . Epidemiology and clinical features of rotavirus and norovirus infection among children in Ji’nan, China. Virology journal 10, 302 (2013). 24099150 10.1186/1743-422X-10-302 PMC3851746

[b26] Simmonds M. K. . New oligonucleotide primers for P-typing of rotavirus strains: Strategies for typing previously untypeable strains. Journal of clinical virology: the official publication of the Pan American Society for Clinical Virology 42, 368–373 (2008). 18378188 10.1016/j.jcv.2008.02.011

[b27] Vinje J., Hamidjaja R. A. & Sobsey M. D. Development and application of a capsid VP1 (region D) based reverse transcription PCR assay for genotyping of genogroup I and II noroviruses. Journal of virological methods 116, 109–117 (2004). 14738976 10.1016/j.jviromet.2003.11.001

[b28] Zhang X. F. . An outbreak caused by GII.17 norovirus with a wide spectrum of HBGA-associated susceptibility. Sci Rep 5, 17687 (2015). 26639056 10.1038/srep17687 PMC4671059

