# Supplementary material for: P[8] and P[4] Rotavirus Infection Associated with Secretor Phenotypes Among Children in South China
Source: Sci Rep. 2016 Oct 6;6:34591. doi: 10.1038/srep34591 (PMC5052604; doi:10.1038/srep34591)
Supplement: Supplementary Information [file srep34591-s1.pdf]

P[8] and P [4] Rotavirus Infection Associated with Secretor Phenotypes Among Children in South China

Xu-Fu Zhang[1]†, Yan Long[1]†, Ming Tan[3,4], Ting Zhang[2], Qiong Huang[5], Xi Jiang[3,4], Wen-Fang Tan[1], Jian-Dong Li[2], Gui-Fang Hu[2], Shixing Tang[2], Ying-Chun Dai [2,*]

Supplement Table 1: Study population demographics

Group	Sample collection	No. of male	No. of female	Range of age	Median of age
**Cases:** RV infection GE patients	Stool sample Saliva	44	31	3 month-10 years old	1.8 years old
**Internal control:** RV/NoV negative GE patients	Stool sample Saliva	89	62	2 month- 9 years old	2.1 years old
**Healthy control:** children in a primary school	Saliva	98	71	6-9 years old	6.7 years old
**Serological study:** 206 persons (103 from Magong and 103 from Hongsao districts	Saliva Serum	78	128	3-88 years old	51.2 years old
